# Altered baseline brain activity in experts measured by amplitude of low frequency fluctuations (ALFF): a resting state fMRI study using expertise model of acupuncturists

**DOI:** 10.3389/fnhum.2015.00099

**Published:** 2015-03-19

**Authors:** Minghao Dong, Jun Li, Xinfa Shi, Shudan Gao, Shijun Fu, Zongquan Liu, Fanrong Liang, Qiyong Gong, Guangming Shi, Jie Tian

**Affiliations:** ^1^Engineering Research Center of Molecular and Neuro Imaging of Ministry of Education, School of Life Science and Technology, Xidian UniversityXi’an, SAA, China; ^2^School of Electronic Engineering, Xidian UniversityXi’an, SAA, China; ^3^The 3rd Teaching Hospital, Chengdu University of Traditional Chinese MedicineChengdu, SC, China; ^4^Department of Radiology, Huaxi MR Research Center, West China Hospital of Sichuan UniversityChengdu, China; ^5^Intelligent Medical Research Center, Institute of Automation, Chinese Academy of SciencesBeijing, China

**Keywords:** resting state functional MRI, expertise, baseline brain activity, functional plasticity, amplitude of low frequency fluctuation, acupuncturist

## Abstract

It is well established that expertise modulates evoked brain activity in response to specific stimuli. Recently, researchers have begun to investigate how expertise influences the resting brain. Among these studies, most focused on the connectivity features within/across regions, i.e., connectivity patterns/strength. However, little concern has been given to a more fundamental issue whether or not expertise modulates baseline brain activity. We investigated this question using amplitude of low-frequency (<0.08 Hz) fluctuation (ALFF) as the metric of brain activity and a novel expertise model, i.e., acupuncturists, due to their robust proficiency in tactile perception and emotion regulation. After the psychophysical and behavioral expertise screening procedure, 23 acupuncturists and 23 matched non-acupuncturists (NA) were enrolled. Our results explicated higher ALFF for acupuncturists in the left ventral medial prefrontal cortex (VMPFC) and the contralateral hand representation of the primary somatosensory area (SI) (corrected for multiple comparisons). Additionally, ALFF of VMPFC was negatively correlated with the outcomes of the emotion regulation task (corrected for multiple comparisons). We suggest that our study may reveal a novel connection between the neuroplasticity mechanism and resting state activity, which would upgrade our understanding of the central mechanism of learning. Furthermore, by showing that expertise can affect the baseline brain activity as indicated by ALFF, our findings may have profound implication for functional neuroimaging studies especially those involving expert models, in that difference in baseline brain activity may either smear the spatial pattern of activations for task data or introduce biased results into connectivity-based analysis for resting data.

## Introduction

Dynamic changes take place in the human brain in response to training throughout the lifespan (Pascual-Leone et al., 2005). Currently, learning-induced neural plasticity has become one of the most challenging topics in modern neuroscience (Lee et al., 2010). Intensive training and deliberate practice leads to expertise (Ericsson et al., 2006). In the last few decades, studies on human brain neuroplasticity using experts models, such as chess masters, meditation practitioners (Brefczynski-Lewis et al., 2007), professional athletes (Park et al., 2009), musicians (Zatorre et al., 1998) and radiologists (Harley et al., 2009), have shown that expertise modulates brain response patterns under task state (Cheng et al., 2007; Erickson et al., 2007; Tang et al., 2007). More recently, researchers have realized that plastic functional representations in response to intensive training may also be present in the resting state (Albert et al., 2009; Lewis et al., 2009).

Coherent spontaneous low-frequency fluctuations in 0.01–0.1 Hz in the blood oxygen level-dependent (BOLD) signal are thought to reflect gross cortical excitability and neuronal synchronization(Logothetis et al., 2001; Balduzzi et al., 2008) and more importantly, play a pivotal role in maintaining the ongoing, internal representations in the coding of prior experience at rest (Lewis et al., 2009). Former research on expertise reported changed connectivity patterns/strength within or between resting brain networks in the experts’ resting brains (Luders et al., 2011; Herholz and Zatorre, 2012; Taylor et al., 2013). These studies have left a question open whether or not expertise modulates baseline brain activity. We suggest that it be a fundamental issue, because the changes in baseline brain activity may either smear the spatial activation under task (Di et al., 2013a) or bring biased results into the connection-based analysis in resting data (Di et al., 2013b).

The amplitude/power (square of amplitude) of low frequency fluctuations in BOLD signals can provide both the nature and extent of signal changes underlying spontaneous neural activity (Fransson, 2006; Duff et al., 2008). The amplitude of low frequency fluctuation (ALFF) is a reliable and reproducible method to detect baseline brain activity in both healthy participants (Yang et al., 2007; Zuo et al., 2010b) and patients (Huang et al., 2010; Qi et al., 2012). Several studies employed it to measure the alterations in baseline brain activity for both healthy subjects (Zuo et al., 2010a) and subjects with pathological conditions (Liu et al., 2012). On the other hand, acupuncturists consist of a novel and robust expertise model for their proficiency in tactile and emotion regulation domains (Dong et al., 2013) as the result of long-term experience. Specifically, for the tactile domain, the exceptional tactile discrimination ability for the functioning digits is essential, because it enables acupuncture practitioners to distinguish subtle dynamic changes of manipulation sensation transmitted through fine acupuncture needles. For the emotion regulation domain, acupuncture inflicts painful treatment procedures in patients, which would induce empathic pain in acupuncturists. Massive exposure to such negative stimuli would naturally induce negative emotions or personal distress in acupuncturists (Cheng et al., 2007), therefore, emotion regulation mechanism, i.e., cognitive reappraisal (Cheng et al., 2007) or altered emotional awareness which is rooted in increased interoceptive signaling triggered by automatic bodily sensations (Lutz et al., 2009; Kirk et al., 2011; Gu et al., 2013), is indispensable to prevent these negative emotions from impairing their professionalism.

Therefore, in the current study, we used ALFF as the metrics of baseline brain activity to determine whether or not expertise modulates local baseline brain activity. Given that resting state activity actively and selectively processes prior experiences (Albert et al., 2009), we expected to see differences in regions which are likely to relate to their behavioral expertise. Furthermore, we conducted a correlation analysis to investigate whether or not such alterations were related to their behavioral expertise.

## Materials and Methods

All research procedures were approved by the West China Hospital Subcommittee on Human Studies and were conducted in accordance with the Declaration of Helsinki. Written informed consent was obtained from all subjects after the experimental procedures were fully explained.

### Subjects

In the preliminary screening session, potential participants underwent a preliminary face-to-face interview and were excluded if they had any current or previous psychiatric or neurological disorders, consumed any psychotropic drugs, or had any severe medical conditions. The basic inclusion criterion for acupuncturists was that they had at least 4 consecutive years of work experience as acupuncturists. The following inclusion criteria were met: (1) right handed (Oldfield, 1971); (2) graduated from national medical schools; and (3) carrying valid licenses for acupuncture practice. Then, participants were asked to fill out a series of self-reported dispositional measures, including the situational pain questionnaire (SPQ) that assessed sensitivity to pain (Clark and Yang, 1983), the Emotional Contagion Scale (ECS) that measured the susceptibility to others’ emotions (Doherty, 1997), and the interpersonal reactivity index (IRI; Davis, 1994). The acupuncturists were from local hospitals. After the preliminary screening session, they were included for a scanning session and post-scanning behavioral expertise tests (*detailed procedure is provided in the “Behavioral expertise” section*). To obtain a cohort of behaviorally homogenous subjects, those subjects whose behavioral expertise fell in the range of (mean ± 2 standard deviations (SD)) were used for this study. Eventually, functional data from 23 healthy right-handed professional acupuncturists were selected for analysis (age: 27.5 ± 1.7 yrs mean ± standard deviation, 10 males, education 20.6 ± 1.6 yrs). Their experience as acupuncturists was an average of 65.5 ± 12.7 months.

The group of non-acupuncturists (NA) consisted of 23 healthy subjects of similar age, handedness and self-report dispositional measures (age: 27.1 ± 1.5 yrs, 10 males, education 20.1 ± 1.3 yrs). The participants consisted of staff in the administrative office and students from non-medical departments from local universities. They also filled out a series of self-reported dispositional measures as mentioned above. The inclusion criteria for NA were: (1) no previous experience working under clinical conditions in the past 4 years; (2) no previous experience of attending any acupuncture lectures or any lectures in Traditional Chinese Medicine; and (3) no experience in acupuncture practice or had no exposure to acupuncture treatment.

### Behavioral Expertise Tests

Acupuncturists’ level of expertise was measured by two tasks. Specifically, the subjects’ tactile discrimination ability for the right index finger and thumb and emotion regulation ability in response to empathic pain were examined. In general, the subjects were seated comfortably in a quiet room with minimal distraction from their surroundings. After the requirements of the tasks were explained to the subjects by the same experimenter, the subjects were required to repeat the procedures and demands of the tasks to the experimenter to ensure all details were explicitly comprehended. These tests did not begin until the experimenter was sure that all procedures were precisely understood by all of the participants. To obtain a comparatively homogenous expert performance, we selected top performers from both dimensions and made sure that the behavioral measurement was in the range of mean ± 2 SD. NA group also went through these behavioral tests.

#### Test on Tactile Discrimination Ability

Tactile spatial acuity is a reliable indicator for the function of the somatosensory system. Previous studies have proven that tactile spatial acuity is highly correlated with subjective tactile perceptual integrity in a broad range of subject populations (Johnson and Phillips, 1981; Johnson and Hsiao, 1992; Van Boven and Johnson, 1994). A well-established and reproducible measure of tactile spatial acuity is the psychophysical spatial discrimination threshold (SDT) for the grating orientation discrimination task (Johnson and Phillips, 1981; Van Boven and Johnson, 1994; Sathian and Zangaladze, 1996). SDT was assessed using Johnson–Van Boven–Phillips domes (Med-Core, St. Louis) at the fingertip (Sathian and Zangaladze, 1996).

In the current study, we used the grating orientation discrimination task to evaluate the subjects’ tactile discrimination ability of the right index finger and thumb. The fingers to be tested were immobilized through double-sided tape affixed to the dorsal aspect of the finger and floor of the fixture to prevent exploratory movements. Grating domes were pressed manually onto the palmar surface of the distal phalanx of the thumb and index finger of the right hand using a spring-loaded apparatus with a spring-loaded force of 1.5 ± 0.2 N, which was adapted following the procedure of a former study (Van Boven et al., 2005). Gratings were applied with the ridges oriented either along or across the long axis of the finger and subjects verbally reported the orientation of the grating as “along” or “across”. During this experiment, subjects were blindfolded and seated comfortably. The experimenter was visually cued to manually position (Van Boven et al., 2005), then released the gratings to the fingertip and maintained static for 1 s as signaled by a computer-driven timing mechanism (Zhang et al., 2005). The subsequent experimental block on each finger consisted of 40 trials without feedback. Participants received a 15 s break after every 20 trials, and a 1 min break between fingers. Each trial consisted of two sequential stimulus presentations (inter-stimulus interval, 2 s) with gratings of identical groove width but differing 90° in orientation, i.e., either parallel (vertical) to or transverse (horizontal) to the long axis of the finger. The stimulus order was chosen randomly. Subsequently, for each finger of each subject, dome gratings with progressively finer spatial periods were used until performance was at or below threshold levels (75% correct responses) (Van Boven et al., 2000, 2005; Goldreich and Kanics, 2003). The grating sizes yielded the SDTs for two fingers of each individual. Lower SDT indicates better tactile proficiency. The SDT was the outcome for this task.

#### Test on Emotion Regulation Proficiency

This test aimed to assess the acupuncturists’ emotion regulation proficiency. A visual stimuli rating task was employed, which was similar to a previous study (Cheng et al., 2007). The unpleasantness ratings were the outcomes for this task. Manipulating acupuncture naturally leads to empathic pain, which would cause a negative emotional response, such as unpleasantness or distress (Hein and Singer, 2008). There must be a certain centrally regulatory mechanism which helps them cope with adverse daily events and prevents negative emotions from impairing their ability to heal or be of assistance (Cheng et al., 2007). Given that “*unpleasantness felt by individuals is the gross production of all cognitive, developmental and social emotion regulatory procedures, and thus, reliably reflected the emotion regulation ability of human beings* (Vogt, 2005)”, we used unpleasantness ratings to index emotion regulation proficiency.

All subjects were shown 120 visual stimuli (120 jpeg files). These stimuli consisted of pictures of different body parts of both painful and neutral situations (Cheng et al., 2007). Pictures were scenarios encountered in daily clinical practice. In half of the stimuli, the body parts were touched by a Q-tip (non-painful situations), and in the other half they were pricked by an acupuncture needle (painful situations). All body parts were chosen to be appropriate acupuncture sites with the assistance of an acupuncture physician with over ten years of clinical experience. The visual stimuli were delivered using the computer program E-prime 2.0 (Psychology Software Tools, Inc.). The sequence of images was randomized. Each image was displayed for 4 s, and rating for unpleasantness lasted 2 s. Participants were asked to focus on the images shown on the screen and began to score only after the cue for scoring appeared. The screen for scoring read “*How intense is the unpleasantness felt by you now?*” Participants responded on a 10-point Likert scale ranging from 1 to 10, where 10 referred to extreme unpleasantness, and 1 referred to no effect. The ratings for unpleasantness were averaged across neutral and negative stimuli respectively for each subject, rather than variations in body parts that were stimulated.

### MRI Data Acquisition

Imaging data were collected using a 3T Siemens scanner (Allegra, Erlangen, Germany) at the HuaXi MR Research Center, West China Hospital of Sichuan University, Chengdu, China. A standard birdcage head coil was used, along with restraining foam pads used to minimize head motion and to diminish scanner noise. After a localizer scan and conventional structural imaging, resting-state functional images were obtained with an echo-planar imaging (EPI) sequence during 6 min and 10 s scanning (185 volumes total, 33 contiguous slices with a slice thickness of 4 mm; TR = 2000 ms; TE = 30 ms; flip angle, 90°; field of view, 240 × 240 mm^2^; data matrix, 64 × 64). During the resting scan, subjects were instructed to keep their eyes closed, not to think about anything and stay awake during the entire session. After scanning, the subjects were asked whether they remained awake during the whole procedure. The axial 3D T1-weighted images were obtained using an MPRAGE sequence with the following parameters: TR = 1900 ms; TE = 2.26 ms; flip angle = 90°; in-plane matrix resolution = 256 × 256; slices = 176; field of view = 256 × 256 mm^2^; voxel size = 1 × 1 × 1 mm^3^. The structural images were used to exclude the possibility of clinical abnormalities by two expert radiologists. None of the participants showed brain abnormalities on conventional MRI.

### MRI Data Preprocessing

Data preprocessing procedures were carried out using Statistical Parametric Mapping (SPM8)^1^ and Data Processing Assistant for Resting-State fMRI (DPARSF) V2.1 Basic Edition (Chao-Gan and Yu-Feng, 2010).^2^ The first 10 volumes were discarded to eliminate non-equilibrium effects of magnetization and allow the participants to adapt to the EPI scanning environment. The images were corrected for the acquisition delay between slices, aligned to the first image of each session for motion correction, spatially normalized and then resampled to 3 mm isotropic voxels. No subjects had head motions exceeding 1 mm of movement or 1°of rotation in any direction. After this, the functional images were spatially smoothed with a 6 mm full width at half maximum Gaussian kernel. Finally, the linear trend was removed and temporal filtering (0.01–0.08 Hz, (Biswal et al., 1995; Lowe et al., 1998)) were performed on the time series of each voxel to reduce the effect of low-frequency drifts and high-frequency noise. The ALFF analysis was carried out using the REST package,^3^ which has been described in previous studies (Yang et al., 2007; Zang et al., 2007). Briefly, filtered time series was transformed to the frequency domain using the fast Fourier transform (FFT). Since the power of a given frequency was proportional to the square of the amplitude of this frequency component, the square root was calculated at each frequency of the power spectrum and the averaged square root was obtained across 0.01–0.08 Hz at each voxel. This averaged square root was taken as the ALFF. For standardization, the ALFF of each voxel was divided by the global mean ALFF value for each subject, resulting in a relative ALFF. The standardized ALFF of each voxel then had a value of about 1, as done in PET studies (Raichle et al., 2001).

### Statistical Analysis

#### Between-Group ALFF Analysis

For the between group analysis, a two-sample *t*-test was performed to detect the ALFF difference between the two groups. The results were considered significant above a threshold of *p* < 0.05, family-wise error (FWE) corrected.

#### Correlation Analysis

To investigate the relationship between ALFF and behavioral measurements in the acupuncturists, we computed the voxel-wise Pearson’s correlation coefficients between ALFF and behavioral measurement (SDT, the unpleasantness ratings and duration of experience). At the level of significance, we applied *p* < 0.05, corrected for the multiple correction at the cluster level, with an underlying threshold of *p* < 0.001 uncorrected at the voxel level. For multiple comparisons, Monte Carlo simulations were performed using the AFNI AlphaSim program.

## Results

### Results of Behavioral Tasks

#### Results of Tactile Discrimination Ability Test

**Figure 1:**
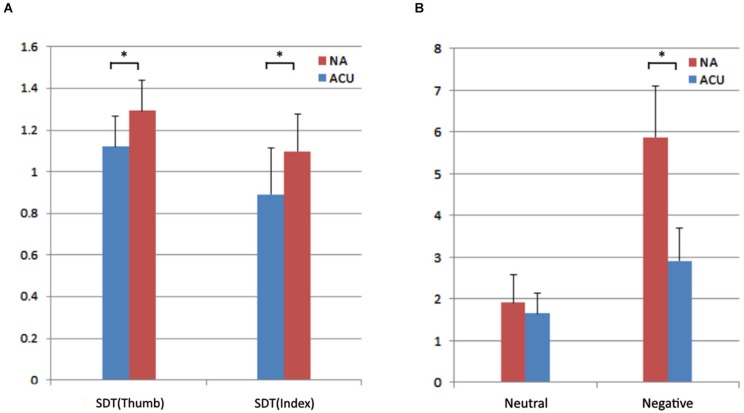
**Results of the behavioral analysis. (A)** Results of tactile discrimination ability test. Acupuncturists had a significantly lower SDT than that of the control group for both fingers (*p < 0.001 and p = 0.001 for the thumb and the index finger respectively, Mann-Whitney test*). **(B)** Results of the emotion regulation proficiency test. Unpleasantness ratings for neutral stimuli did not differ in the two groups (*p* = 0.21, *Mann-Whitney test*). Unpleasantness ratings for negative stimuli were significantly lower in the acupuncturist group (*Mann-Whitney test, p < 0.05*). ACU: acupuncturists; NA: non-acupuncturists; * denotes significant difference between groups.

As shown in Table 1, acupuncturists had a significantly lower SDT than that of the control group for both fingers (*p < 0.001 and p = 0.001 for the thumb and the index finger respectively, see* Figure 1A; Table 1 *for details, Mann-Whitney test*), indicating better spatial acuity in the group of acupuncturists. The results proved the acupuncturists’ tactile discrimination proficiency. Mann-Whitney test was used because the data did not meet the assumptions of a Student’s *t*-test.

**Table 1:** **Psychophysical measurement of tactile ability in the acupuncturist and NA groups**.

	Expert (*n* = 23)		Controls (*n* = 23)		Mann-Whiteney U-Test (Experts vs. NA)	
Task	Mean	SD	Mean	SD	*p*-value	*Z*-value
^∧^SDT*(Thumb)	1.12	0.15	1.29	0.14	<1E-3	−3.499
^∧^SDT*(Index)	0.89	0.22	1.1	0.18	0.001	−3.288

* denotes the item that shows a significant difference between groups. p < 0.05.

*∧ denotes that Mann-Whitney test was used*.

#### Results of the Emotion Regulation Proficiency Test

The analyses of the dispositional measures revealed no differences between the two groups in terms of ECS, SPQ and each sub-domain of IRI scores using the *Mann-Whitney test and two sample t-test* (*p* > 0.05, see Table 2 *for details*). Detailed information is summarized in Table 2. Unpleasantness ratings for neutral stimuli did not differ in the two groups (*p* = 0.21, Mann-Whitney test, Figure 1B; Table 2) as expected, whereas for negative stimuli, unpleasantness ratings were significantly lower in the acupuncturist group (*Mann-Whitney test, p* < 0.05, Figure 1B; Table 2). The results demonstrated that compared with the NA group, the acupuncturists had better emotion regulation ability. Mann-Whitney test was used because the data did not meet the assumptions of a Student’s *t*-test.

**Table 2:** **Dispositional measurement of empathy and ratings of unpleasantness in the acupuncturist and NA groups**.

	Expert (*n* = 23)		Controls (*n* = 23)		Mann-Whiteney U-Test (Experts vs. NA)	
Task	Mean	SD	Mean	SD	*p*-value	*Z/t*-value
**^∧^ECS**	29.6	13.7	31.7	15.3	0.621	−0.499
**^∧^SPQ**	5.4	0.8	5.5	0.9	0.813	−0.273
**^∧^IRI(PT)**	16.5	2.9	17.3	3.1	0.579	−0.566
**#IRI(EC)**	19.2	3.4	20.3	2.9	0.2526	−1.1593
**^∧^IRI(PD)**	12.5	4.9	13.3	3.7	0.332	−0.982
**^∧^IRI(FS)**	17.5	3.6	16.8	4.3	0.619	−0.507
**^∧^Unpleasantness (Neutral)**	1.7	0.5	1.9	0.7	0.212	−1.348
^∧^Unpleasantness*(Negative)	2.9	0.8	5.9	1.2	<1E-3	−5.881

Abbreviations: emotional contagion scale (ECS), situational pain questionnaire (SPQ), interpersonal reaction index (IRI), perspective taking (PT), empathic concern (EC), personal distress (PD) and fantasy (FS); standard deviation, SD;

*denotes the item that shows significant difference between groups. p < 0.001.

*∧denotes that Mann-Whitney test was used*.

*#denotes that two-sample t-test was used*.

### Results of MRI Data

#### Between-Group ALFF Results

**Figure 2:**
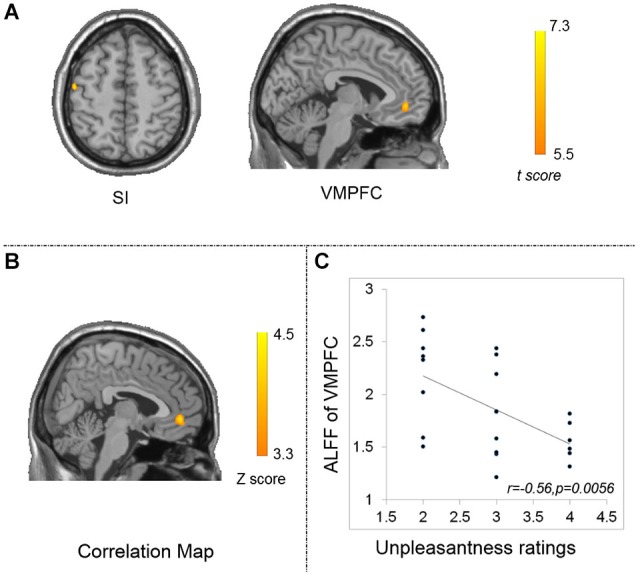
**Results of ALFF differences between groups (*p* < 0.05, FWE corrected) (A) and correlation maps between ALFF and unpleasantness ratings for the acupuncturist group (B,C). (A)** the acupuncturist group showed larger ALFF in the left VMPFC (displayed in sagittal view) and the left SI (displayed in axial view). **(B)** Significant correlation between ALFF and unpleasantness ratings was found in the left VMPFC. The statistical threshold was set at |Z| > 3.29 (*p < 0.001* at the individual voxel level) and cluster size >45 voxels, which corresponds to a corrected *p* < 0.05. **(C)** scatter plot map computed as ALFF of the peak voxel in the correlation analysis and unpleasantness ratings. VMPFC: the ventral medial prefrontal cortex; SI: the primary somatosensory cortex.

The results of the two-sample *t*-test (*p* < 0.05, FWE corrected) demonstrated a higher ALFF value in the left ventral medial prefrontal cortex (VMPFC) and hand representation of primary somatosensory area (SI), contralateral to the pro-hand in the acupuncturists (Figure 2A; Table 3). The location is consistent with loci of SI reported in previous studies on hand-finger representation (Tegenthoff et al., 2005; Lissek et al., 2009). No brain regions with a significant ALFF decrement were found.

**Table 3:** **Significant group ALFF differences for the acupuncturist group (p_FWE_ < 0.05)**.

	Hemisphere	MNI Coordinates (cluster maxima)			Voxels	t (cluster maxima)
		*x*	*y*	*z*
VMPFC	L	−4	50	−8	29	6.9
SI	L	−48	−12	53	16	7.29

*VMPFC: ventral medial prefrontal cortex; SI: primary somatosensory area*.

### Results of the Correlation Analysis

Significant negative correlation between ALFF and the unpleasantness ratings was found in the left VMPFC correlated with the acupuncturist group (*Alphasim correction*, the corresponding statistical level is set at |Z| > 3.29 (*p* < 0.001 at individual voxel level) and cluster size > 45 voxels (*search volume* = 70831 *voxels*)), which corresponds to a corrected *p* < 0.05 (Figure 2B). Its peak voxel (−5, 44, −5; *Z* = 4.42) was located within the left VMPFC. We display a scatter plot between unpleasantness ratings and the peak ALFF voxel from the correlation map (*r* = −0.56, *p* = 0.0056) (Figure 2C). No such correlations were found between outcomes of other behavioral tests and ALFF, as well as between the length of acupuncture practice and ALFF for the acupuncturist group exceeding the level of significance (*p* < 0.05, corrected for the multiple corrections at the cluster level, with an underlying threshold of *p* < 0.001 uncorrected at the voxel level). No such correlations were found between results of the behavioral tests and ALFF for the non-acupuncturist group exceeding the level of significance.

## Discussion

Recently, MRI studies employing expertise models have attracted a lot of scientific attention. But, most of these studies have ignored a rather fundamental issue whether or not the baseline level brain activity is altered with expertise. We used ALFF as the metric to measure the level of baseline brain activity and the expertise model of highly trained professional acupuncturists to look into the above mentioned question. For the behavioral data, the group of acupuncturists outperformed the group of NA (Figure 1; Tables 1, 2). For the MRI data, higher ALFF values were identified in the left VMPFC and the contralateral hand representation of SI for the acupuncturist group (Figure 2A; Table 3). Negative correlation between the outcomes of emotion regulation task and ALFF were found in the left VMPFC. Our results showed that experts may have different baseline brain activity, i.e., altered amplitude of low frequency fluctuation contrasted with laypersons. Taken that resting state actively processes prior experience (Miall and Robertson, 2006), we speculated that these changes may code the learned experience, serving a role in skill maintenance (Peigneux et al., 2006).

Tactile discrimination ability is essential for acupuncture practitioners. Acupuncture aims to elicit the signified sensation in patients, which is characterized as subtle but unique tightness around the fine acupuncture needle to achieve optimal therapeutic effects (Cheng, 2010). Patients’ bodily response is dynamic since their bodily state and psychophysiological state is constantly changing during each round of needle rotation. Therefore, in acupuncturists’ daily manipulation, it is highly needed to distinguish subtle and dynamic changes of manipulation sensation on the fingers, which is transmitted through the fine needle to determine whether the target sensation is obtained. On one hand, our behavioral test demonstrated that acupuncturists’ had a lower individual spatial discriminative threshold, indicating better tactile perception (Table 1), which would facilitate the acupuncturists’ tactile discrimination ability. On the other hand, our results revealed higher ALFF values in the contralateral SI (Figure 2; Table 3). This region is responsible for the perception of touch (Frackowiak, 2004). Training has been shown to result in better stimulus-specific sensory processing (Snowden et al., 2000), which is quite likely to be the consequence of improved recognition or classification of the stimuli (Johnson, 2003). Previous studies have shown that the increase in tactile performance is paralleled with neuroplastic changes in this region either as a result of extensive training/learning (Jäncke, 2009) or a result of enriched sensory input (Pleger et al., 2003). For acupuncturists, extensive training is required for skill acquisition and, in return, it induces increased sensory input. Given that extensive training/learning and enriched sensory input would induce improvement of tactile perception ability and drive functional plastic changes (Lewis et al., 2009; Lissek et al., 2009), we proposed that the alterations in this region may be associated with acupuncturists’ tactile proficiency. The results of PET studies showed that sensorimotor learning induced cerebral blood flow increase in the resting human brain (Xiong et al., 2009), indicating excitability in neuronal activities. ALFF has been suggested to be related to cortical excitability and human cognitive performance (Fransson, 2006; Duff et al., 2008). Moreover, a recent study reported that ALFF from the resting state data was correlated with cerebral blood flow in the corresponding regions (Li et al., 2012). Taken together, we suggest that higher ALFF is likely to be associated with learning and clustered changes in this region, i.e., higher ALFF values in the contralateral SI may support the specialization of sensory cortices for perceptual awareness of a specific modality (Boly et al., 2007), which may facilitate acupuncture’s increased processing of sensory stimuli (Johnson, 2003; Dong et al., 2014).

Acupuncturists inflict painful treatment procedures in their daily practice. This procedure would naturally induce empathic pain. Interestingly, acupuncturists gave significantly lower unpleasantness ratings than NA in the behavioral task (Table 2). The difference is not likely to be attributed to dispositional variables such as sensitivity to pain, empathy disposition, or emotion contagion, because the two groups did not differ in these traits (Table 2). Massive exposure to empathic pain would induce personal distress and eventually hamper their ability of being of assistance (Cheng et al., 2007). Therefore, emotion regulatory skills, which are likely derived from acupuncturists’ professional knowledge of acupuncture, must operate to prevent negative emotions from impairing their capacity of assistance, although they may not be aware of this process (Cheng et al., 2007). Accordingly, we suggest that the differences in unpleasantness ratings may be due to a certain centrally modulatory mechanism in acupuncturists that prevents negative emotions from jeopardizing their professional abilities. In our study, higher ALFF in the left VMPFC was identified (Figure 2; Table 3). This is in line with the findings detecting acupuncturists’ emotional responses under task (Cheng et al., 2007). VMPFC participates in emotion regulation, and specifically, it is actively engaged in suppressing or reappraising negative emotional stimuli and was also reported to play a role in suppressing the influence of negative emotional stimuli on subsequent behavior (Quirk and Beer, 2006). This region has shown plastic changes in the context of emotion regulation (Davidson et al., 2000). Additional support regarding the role of this region is the correlation between ALFF values and outcomes of the emotion regulation task (Figures 2B,C). Therefore, we propose that this region may contribute to emotion regulation proficiency in acupuncturists. In the resting state, a former study focusing on how learning influences inter-region connectivity patterns has demonstrated that acquisition of a cognitive skill after long term training would cause the optimization of inter-regional communication efficiency (Bassett et al., 2011). Critically speaking, concomitant with overt behavioral adaptation, conversancy (the acquaintance with a behavior, especially as a result of study or experience) in behavioral performance is naturally supported by the aggregated subsystems/regions without perturbing the remainder of the system, implicating a cost-efficient central mechanism (Bassett et al., 2009). It is probable that the clustered changes in a local area, i.e., higher ALFF in the left VMPFC, are consistent with this idea and may reflect this tendency that may facilitate acupuncturists’ expertise.

Cognitive reappraisal (Cheng et al., 2007) and altered emotional awareness which is rooted in increased interoceptive signaling triggered by automatic bodily sensations (Lutz et al., 2009; Kirk et al., 2011; Gu et al., 2013) may serve as candidate mechanisms behind emotion regulation. In general cases for physicians, such as surgeons, dentists and acupuncturists, they all have to inflict pain in the course of their treatment (Decety et al., 2010). However, their experience and professional knowledge instructs them this process is necessary for the ultimate cure. Therefore, reappraisal, that is reinterpreting the meaning of such events and eventually alter its emotional impact (Gross, 1998), may yield facilitation in emotion regulation. For our specific expertise model of acupuncturists, acupuncturists may have to calm themselves to attentively discriminate tactile input, during the treatment process by regulating their autonomic response, such as respiration rate, heart rate etc., which leads to interoceptive signaling. The increased interoceptive awareness may also lead to altered emotional response (Gu et al., 2013). The central representation of the reappraisal process is likely to be the VMPFC (Gross, 1998; Cheng et al., 2007) and the insular for the interoceptive signaling (Craig, 2002; Critchley et al., 2004). Given the engagement of VMPFC and disengagement of the insular, combined with a recent conclusion that interoceptive awareness facilitates reappraisal (Füstös et al., 2013), we suggest that the cognitive reappraisal may serve as the potential mechanism behind acupuncturists’ emotion regulation proficiency.

Our current findings may have further implications especially in the context that neuroimaging studies using expert models have introduced promising findings both under task and resting state. For functional studies employing tasks, findings from Biswal’s group revealed that the alterations in amplitude of the BOLD signal, as indicated by ALFF, would smear and distort the spatial activation of the activation maps (Di et al., 2013a); For resting state fMRI studies, alterations in ALFF would serve as a potential confound leading to biased connectivity patterns and locations when studying functional connectivity and networks (Di et al., 2013b). Combined together, we suggest that alterations in baseline level brain activity should be taken into consideration particularly for functional MRI studies employing expertise models.

At last, we suggest that several limitations should be taken into consideration when interpreting the current findings. First, as in all cross-sectional studies, confounding factors such as genetic predispositions or training in critical periods during development cannot be ruled out as possible explanations for the observed differences between groups, so we cannot yet determine whether the observed differences are actually acquired through training, or whether they are simply the result of a rare phenotype within the general population. Future studies should consider longitudinal experimental design to further test the hypothesis. Secondly, given the comparatively short sample size, we are inviting the replication of our findings with larger samples to make our conclusion more reliable, although the current study has adopted relatively conservative statistical threshold for the main findings.

## Author’s Contribution

Designed the experiments: MHD, JL. Performed the experiments: MHD, ZQL, WSL. Analyzed the data: LZW, XFS. Contributed reagents/materials/analysis tools: FRL, QYG., JT. Wrote the paper: MHD. Contributed to the writing of the manuscript: GMS.

## Conflict of Interest Statement

The authors declare that the research was conducted in the absence of any commercial or financial relationships that could be construed as a potential conflict of interest.

## Footnotes

^1^http://www.fil.ion.ucl.ac.uk/spm

^2^http://www.restfmri.net/forum/DPARSF

^3^http://resting-fmri.sourceforge.net
